# City and Country

**Published:** 1882-10-20

**Authors:** R. C. Word

**Affiliations:** Man’g Editor


CITY AND COUNTRY.—The October number of this valuable publication comes to our table filled as ever with good things. As illustrations it has "Chock full of Mischief"—a full page plate; "A Festival in a Shanghai Tea Garden," "The Lansing Evaporator," "City and Country Homes," and "The Hansell Raspberry." "Honor's Debt" is concluded in this issue, and a short serial, "A Strange Discovery," is commenced by Miss Josie C. Malott. The editorials cover every ground, and the one on "Politics in Ohio" is able and will be largely quoted. "Articles on Farm Law" by Hon. Edmund H. Bennett still continues. This valuable publication should be a regular visitor at every fireside. Only 50 cents per year with choice of two premiums. Will C. Turner, editor. A. W. Lincoln, associate. CITY AND COUNTRY Co., publishers, Columbus, Ohio.

The *City and Country* will be sent as a premium to any one sending us a new cash subscriber.

R. C. WORD, Man'g Editor.

